# Retraction: Icariin ameliorates palmitate-induced insulin resistance through reducing thioredoxin-interacting protein (TXNIP) and suppressing ER stress in C2C12 myotubes

**DOI:** 10.3389/fphar.2023.1299165

**Published:** 2023-10-06

**Authors:** Frontiers Editorial Office

The journal retracts the 2018 article cited above.

Following publication, concerns were raised regarding the integrity of the images in the published figures. Image duplication concerns were identified in **Figures 1C, 2B, 6C, E, and 7E**. The authors failed to provide a satisfactory explanation during the investigation, which was conducted in accordance with Frontiers’ policies. As a result, the data and conclusions of the article have been deemed unreliable and the article has been retracted.

This retraction was approved by the Chief Editors of Frontiers in Pharmacology and the Chief Executive Editor of Frontiers. The authors have not responded to correspondence regarding this retraction.

